# Revealing the Mechanisms of Synergistic Action of Two Magainin Antimicrobial Peptides

**DOI:** 10.3389/fmedt.2020.615494

**Published:** 2020-12-21

**Authors:** Burkhard Bechinger, Dennis Wilkens Juhl, Elise Glattard, Christopher Aisenbrey

**Affiliations:** ^1^University of Strasbourg/CNRS, UMR7177, Institut de Chimie de Strasbourg, Strasbourg, France; ^2^Institut Universitaire de France (IUF), Paris, France

**Keywords:** PGLa, membrane topology, membrane pore, membrane macroscopic phase, SMART model, carpet model, peptide-lipid interactions, molecular shape concept

## Abstract

The study of peptide-lipid and peptide-peptide interactions as well as their topology and dynamics using biophysical and structural approaches have changed our view how antimicrobial peptides work and function. It has become obvious that both the peptides and the lipids arrange in soft supramolecular arrangements which are highly dynamic and able to change and mutually adapt their conformation, membrane penetration, and detailed morphology. This can occur on a local and a global level. This review focuses on cationic amphipathic peptides of the magainin family which were studied extensively by biophysical approaches. They are found intercalated at the membrane interface where they cause membrane thinning and ultimately lysis. Interestingly, mixtures of two of those peptides namely magainin 2 and PGLa which occur naturally as a cocktail in the frog skin exhibit synergistic enhancement of antimicrobial activities when investigated together in antimicrobial assays but also in biophysical experiments with model membranes. Detailed dose-response curves, presented here for the first time, show a cooperative behavior for the individual peptides which is much increased when PGLa and magainin are added as equimolar mixture. This has important consequences for their bacterial killing activities and resistance development. In membranes that carry unsaturations both peptides align parallel to the membrane surface where they have been shown to arrange into mesophases involving the peptides and the lipids. This supramolecular structuration comes along with much-increased membrane affinities for the peptide mixture. Because this synergism is most pronounced in membranes representing the bacterial lipid composition it can potentially be used to increase the therapeutic window of pharmaceutical formulations.

## Introduction

Antimicrobial peptides (AMPs) are part of the innate immune system of higher organisms which provides a powerful and responsive first line of defense against a multitude of pathogenic microorganisms (1, 2). Several years after the discovery of penicillin in 1928 (3) gramicidin S was extracted from soil bacteria and used to treat gunshot wounds during world-war II (4–6). Furthermore, other peptidic compounds with antimicrobial activities have been detected in microorganisms (7, 8), but antimicrobial peptides also exist in many species of the plant and animal kingdom, including humans (9). The first of these peptides have been discovered decades ago and have been investigated ever since (4, 10–12). The list of amino acid sequences with antimicrobial activities is continuously increasing and they are accessible through various data bases (13–16). To understand their mechanisms of action several of them have been investigated by a variety of biological, biochemical, and biophysical approaches (17, 18).

During recent years the rapid increase in multiresistant pathogens (19) has brought back research on AMPs (20, 21), because their mechanisms of action has been shown to be less prone to microbial resistance when compared to conventional treatments (2, 22, 23). Although peptides are quickly digested by proteases (24–26) this limitation in their applicability can be overcome by unnatural building blocks, their protection inside nanostructures or when linked to surfaces (27–29). Furthermore, based on mechanistic studies of cationic amphipathic antimicrobial peptides small amphipathic molecules (30, 31), foldamer and pseudopeptides (32–40), and polymers (41) with potent antimicrobial properties have been designed.

Peptides have been found early on in the skin of toads and frogs (10, 42) and magainins from *Xenopus laevis* were among the first, for which the potential usefulness of their antimicrobial activity has been described (Table 1) (11). Soon after their discovery a multitude of investigations have been performed that reveal that magainins and other cationic amphipathic peptides interfere with the barrier function of bacterial membranes which by itself causes bacterial killing (43–45). Notably, related peptides have been shown to also enter the cell interior where further action can take place (46–48). Furthermore, many peptides are involved in modulating the immune response of the host organisms thereby adding an additional layer of efficiency and they are therefore also referred to as “host defense peptides” (49–52).

**Table 1:** Amino acid sequences of selected antimicrobial peptides.

Magainin 1	GIGKF LHSAG KFGKA FVGEI MKS
Magainin 2	GIGKF LHSAK KFGKA FVGEI MNS
PGLa	GMASK AGAIA GKIAK VALKA L-NH_2_
LAH4	KKALL ALALH HLAHL ALHLA LALKK A-NH_2_
LL37	LLGDF FRKSK EKIGK EFKRI VQRIK DFLRN LVPRT ES
Cecropin P1	SWLSK TAKKL ENSAK KRISE GIAIA IQGGP R
Cecropin A	KWKLF KKIEK VGQNI RDGII KAGPA VAVVG QATQI AK-NH_2_
Melittin	GIGAV LKVLT TGLPA LISWI KRKRQ Q-NH_2_

A membrane-active mechanism has been confirmed by the study of all-D analogs of AMPs which exhibit the same activity than their naturally occurring counter-part indicating that they do not interact with chiral proteinaceous receptors (47). Indeed, their amphipathic nature and in most cases an accumulation of cationic residues has been shown essential for membrane interaction and selectivity, rather than a specific amino acid composition (2). Peptides with helical (17, 18), cyclic (40, 53–55) and/or β-sheet arrangements have been investigated (56–60).

Magainins and related sequences have been developed up to phase IIb/III clinical trials (61, 62), and in parallel, have been explored in considerable detail by biophysical approaches [e.g., (63–66)]. The data from these studies were often unexpected and resulted in the need to introduce novel mechanisms to explain the activities of these peptides (67–69). Because the peptides and/or their biophysical investigations have been reviewed recently (20, 21, 70), this paper will only shortly summarize some of the key discoveries made with magainins and then focus on the synergistic interactions between PGLa and magainins. Combining antimicrobial peptides provides an interesting and little exploited alternative strategy to enhance their efficiency and to further reduce their susceptibility to bacterial resistance.

## Magainins Form Membrane Openings

Magainins exhibit pore-forming and lytic activities when added to membranes which have also been studied by single-channel measurements (45, 71). On a macroscopic scale, magainin pore formation was investigated by fluorophore release experiments. For example, this allowed to measure the release kinetics from individual DOPC/DOPG giant unilamellar vesicles (GUV) at different peptide-to-lipid molar ratios (72). While after peptide addition it takes minutes before the release of fluorophores sets-in, once the pores have formed the micrometer vesicles empty within 30 s (73). It has been measured that fluorophore release is a two-stage process starting with the transient formation of very large pores corresponding to the equilibration of the peptide concentration between the outer and inner leaflets (74). Thereafter a slower but persistent release of fluorophore is observed through pores of 3 nm hydrodynamic radius, large enough to allow passage of globular proteins > 20 kDa (74).

The spatio-temporal events when antimicrobial peptides attach to live bacteria have been investigated by microscopic imaging techniques. Interestingly, the human peptide LL37 (Table 1) preferentially attacks septating *E. coli* cells at the septum and at the curved regions of the outer membrane (75). In non-septating cells, the peptide accumulates at one of the endcaps. As expected permeabilization starts with the outer membrane and after a short delay cytoplasmic membrane permeabilization occurs. The openings at both membranes occur in a localized and persistent manner (76). Related events are observed when cationic polymers, longer or shorter peptides such as LL37, cecropin A, or melittin are studied (Table 1) even though the exact details vary with the antimicrobial compound (77). Finally, the peptides enter the cells where they interact with anionic polymers including nucleic acids and proteins that are abundant in the cell interior (46, 48, 78).

## Magainin Structural Investigations

Structural investigations show that magainins undergo a random coil to helix transition when they partition into the membrane (79). Interestingly the energies associated with this refolding have been identified to be one of the main driving forces for membrane association (80). During the same time period when the antimicrobial activity of magainins was investigated solid-state NMR approaches applied to uniaxially oriented lipid bilayers were under development (63). The latter technique measures angular constraints from polypeptides reconstituted into uniaxially oriented phospholipid bilayers to calculate their structure, topology, and dynamics (81–84). Because the ^15^N chemical shift of peptide bonds alone already provides an approximate tilt angle of helical domains (85) the very first experiments with magainin 2 and PGLa were indicative that these helices are oriented parallel to the membrane surface (63, 86–88). A membrane alignment parallel to the lipid bilayer surface has been confirmed for magainin 2 (89), for magainin analogs (90, 91) and for several other linear cationic antimicrobial peptides (22, 92–95). Later on oriented CD spectra and fluorescence quenching experiments confirmed such a topology of the magainin helix where the latter approach also shows an interfacial localization of the magainin 2 helix (64, 96). A parallel alignment of cecropin P1 (Table 1) using ATR FTIR was later on described by the “carpet model” (97). Notably, this peripheral membrane topology assures that the peptides can exchange between the membrane and the aqueous phase (89).

**Figure 1:**
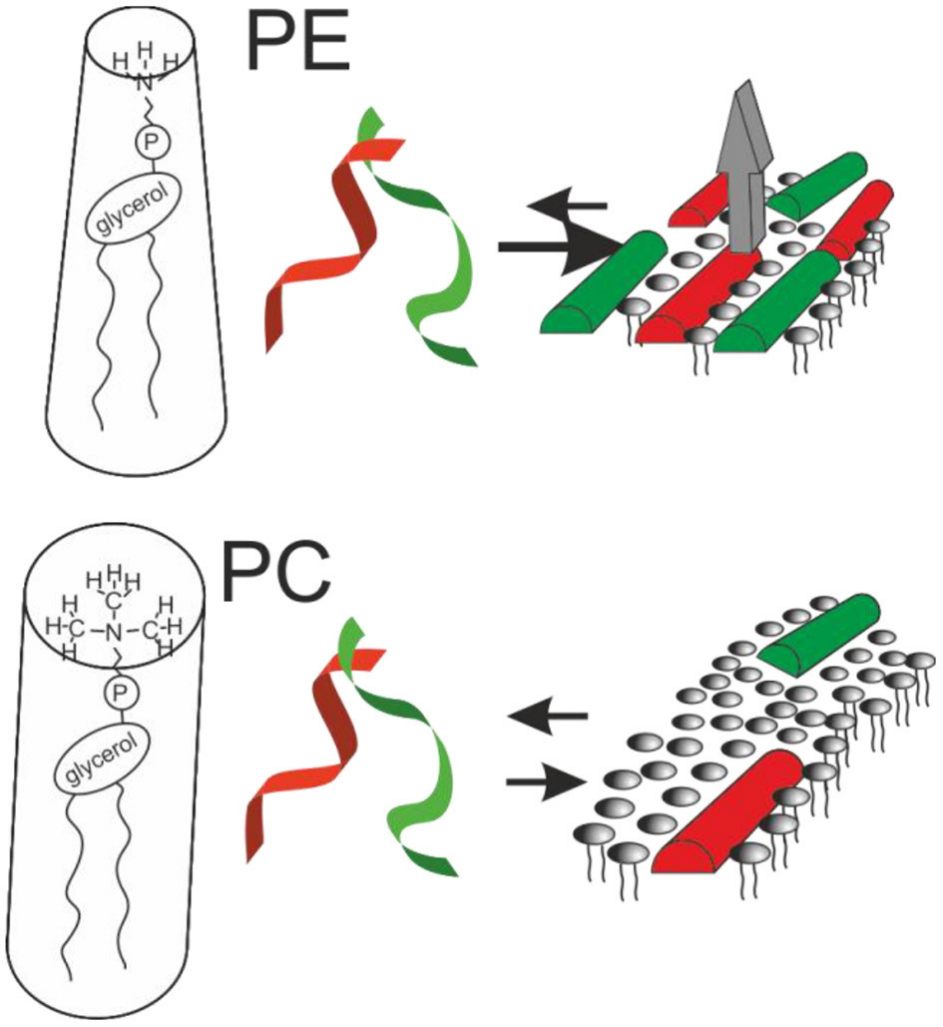
Sketches the structural findings made with magainin 2 (red) and PGLa (green). Both peptides adopt helical conformations that are oriented parallel to the membrane surface in membranes carrying lipid unsaturations. The peptides arrange in nematic mesophases which when added together result in increased membrane affinity and synergistic calcein release activity from POPE-containing liposomes (gray arrow; cf. text for details).

Whereas, the magainin 2 helix has been found to partition into the membrane parallel to its surface regardless of membrane lipid composition (64, 89), its relative PGLa (Table 1) exhibits a much wider range of topologies but only in membranes where all fatty acyl chains are saturated (89, 98, 99). In the presence of magainin 2 PGLa adopts transmembrane alignments (98–101) and early on this configuration has been suggested to be part of a synergistic complex between the two peptides (100, 102). However, in the presence of lipid unsaturations (such as palmitoyl-oleoyl-phosphatidylethanolamine), both PGLa and magainin 2 are aligned along the bilayer surface under all conditions so far investigated (98, 99, 101). Because unsaturations are an integral part of bacterial membranes it is highly likely that both magainin and PGLa exert their antimicrobial activity in a state with their amphipathic helices aligned within the membrane plane [e.g., data obtained with membranes made from *E. coli* lipid extracts (103)]. Correspondingly, alternative models explaining synergism have been suggested (69, 103) (Figure 1).

Notably, recent fluorescence self-quenching investigations have demonstrated the formation of nematic phases at the membrane surface by magainin, PGLa, and the LAH4 amphipathic designer peptide (Figure 1, Table 1). Formation of these supramolecular arrangements is dependent on the lipid composition and the salt concentration of the surrounding buffer suggesting that electrostatic but also lipophobic interactions contribute (69, 104).

An amphipathic peptide that partitions into the membrane interface takes up the place of a few lipids but penetrates only to the depth of the head group and glycerol region (64). Thereby the packing of the hydrophobic region becomes more disordered and the membrane thickness is reduced (105, 106). The resulting changes of the macroscopic phase properties, the bilayer packing and the dynamics of the lipids has been monitored by ^2^H and ^31^P solid-state NMR spectroscopy (107). Thereby magainin peptides have been shown to introduce curvature into membranes (106), to cause a considerable decreases in the fatty acyl chain order parameters in particular of lipid segments well into the bilayer interior (101, 108–110), and at higher peptide concentrations membrane disruption into micellar or bicellar structures (111). Such bilayer disordering has been estimated to reach over a diameter of 10 nm (112, 113).

Atomistic views how in-plane oriented peptides potentially form water-filled openings in lipid bilayers have been obtained from molecular dynamics simulations (114–116). A reoccurring limitation of the computational approaches is the limited time span covered by the simulations which does not allow to reach equilibrium of statistically relevant numbers or peptides. Therefore, at the present stage the results remain dependent on the starting conditions and careful comparison with experimental data an important control (115–117). Furthermore, for magainins the importance of the very details of the lipid composition has only become apparent about a decade ago (69, 98, 118, 119), therefore, only few such simulations have been performed with lipid compositions such as POPE/POPG 3/1 that closely mimic bacterial membranes. All-atom simulations covering 100 ns show a stable in-plane topology of magainin, some oligomerization, but no pore or supramolecular rearrangement within this time frame (120). Stable in-plane alignments and an interfacial localization in such membranes were also observed in more recent coarse-grain and all-atom simulations (116, 117). In summary, pores rather form through stochastic rearrangements of peptides and lipids rather than well-defined channel structures although some peptide-peptide interactions are sometimes apparent. Because of the relatively small size of the membrane patches and the short time frame covered by the simulations the membrane lytic nature of the peptides or the formation of large pores apparent in dye release experiments do not become visible (73, 74).

The molecular shape concept provides a rationale for the bilayer disruptive properties of in-plane aligned amphipathic helices partitioning into the interface (121, 122). Geometrical considerations originally developed for lipids explain why PC lipids, that have the shape of cylinders, spontaneously arrange into extended bilayers. In contrast, PE exhibits a cone shape with a tendency to adopt hexagonal phases and detergents are inverted cones that arrange into micelles. In an analogous manner, surfactin, a cyclic peptide with a long fatty acyl chain (55), or the magainin 2 in-planar interfacial helix use up much more space in the head group than in the hydrophobic core region of the membrane, therefore, the molecular shape concept has recently been elaborated in some detail for amphipathic antimicrobial peptides (122).

Thus, magainins and other cationic amphipathic antimicrobial peptides appear to fulfill the criteria of the “carpet” model where peptides cover the membrane surface at alignments parallel to the surface (67) ultimately causing membrane lysis (111, 123). At lower peptide-to-lipid ratios the lipid membranes can adapt and compensate for the disruptive properties of the peptides, however from time to time openings form locally and transiently thereby resulting in channel-like recordings (124). The structural changes of the membrane have been monitored in the presence of magainin 2 in real-time revealing intermediate states, lysis and recovery (125). The peptides and lipids show a high degree of structural plasticity thus membranes can adopt a broad range of possible morphologies a behavior that is best described by phase diagrams which can take into account the peptide concentration, the membrane composition and other parameters (126). Such features are summarized in the SMART model where “Soft Membranes Adapt and Respond, also Transiently” (127). In line with this model, AMPs have been proposed to affect the membrane line tension (125, 128).

Magainin and PGLa carry several positive charges (nominal charge +4 to +5) and have been shown to interact better with negatively charged membranes. Such preferential association driven by electrostatic attraction is thought to be a reason for the selective killing of bacteria or tumor cells, that expose negative charges while they do not affect healthy eukaryotic cells which expose an overall charge neutral surface (129–133). Indeed, Joachim Seelig and co-workers have quantitatively dissected the interactions of membrane-association into an attractive electrostatic component and a hydrophobic insertion (with a partitioning constant around of the latter of 1,000 M^−1^) (130, 134). Notably, the apparent membrane association is more than an order of magnitude increased for bilayers mimicking the overall anionic composition of bacterial membranes (66). However, this relatively modest membrane association is boosted by electrostatic attraction to negatively charged surfaces which can dominate the association process (122, 135). Furthermore, the membrane association of multicationic antimicrobial peptides has been suggested to result in the interference of electrostatic attractions that keep peripheral membrane proteins in place and consequently antimicrobial action (68).

## Magainin 2—PGLa Mixtures Show Synergistic Enhancement in Biological Assays

Interestingly combinations of antimicrobial compounds are sometimes much more potent than the individual components (49, 136). These observations have been made with mixtures of peptides (137), of peptides with conventional antibiotics (41, 138–142), with blood plasma components (143), or with ions (144). In particular magainin 2 has been shown to interact synergistically with PGLa (45) and with tachyplesin I a cyclic β-sheet peptide (145).

Because magainin and PGLa peptides (Table 1) are produced together and stored as a cocktail in the skin of *Xenopus laevis* frogs, very early on antimicrobial assays have been performed with mixtures of the two peptides. It was soon realized that the peptides exert cooperative behavior (26, 44, 45, 146, 147). These studies involved *E. coli* (24, 26, 43, 148), mitochondria (43), tumor cells (26), calcein loaded liposomes (26, 45), cytochrome oxidase liposomes (24), and hamster spermatozoa (44). Notably, in the latter case only the mixture exhibits detectable activity at all. More recent papers also report synergism of magainin and PGLa on a few selected bacterial species (*E. coli, S. aureus*, and *S. epidermis*) with somehow varying enhancement factors (102, 103, 119, 149).

In particular, the early investigations revealed the poration of membranes, loss of membrane potential, uncoupling of membranes concomitant with effects on respiratory control and thereby interference with energy production and cell survival [e.g., (26, 44, 45, 146)]. In a most recent investigation it was shown that both PGLa and magainin are capable to form fibers at physiological conditions (150). These fibers are somewhat less active in antimicrobial assays but maintain the synergy. Because the peptides are stored in the granules of the frog skin at high concentrations it was speculated that they form functional amyloids for protease protection and graded release (150).

In further studies these investigations have been extended to variants of the peptides and/or other bacteria and the combination index CI has been used as a quantitative measure of synergism (103, 151):


(1) $$\begin{array}{l} CI=\;\frac{0.5\;MIC_{50}^{a+b}}{MIC_{50}^{a}}+\frac{0.5\;MIC_{50}^{a+b}}{MIC_{50}^{b}}, \end{array}$$

where MIC^i^ is the MIC value determined for peptide i alone (i = a or b), and MIC^a+b^ is the total peptide concentration at the MIC determined for the combination. For values <1 synergistic enhancement occurs whereas values > 1 correspond to antagonism (152). In other work the fractional inhibitory concentration index (FICI) has been used which follows a related definition (102).

In order to analyze bacterial growth curves in a quantitative manner, we have early on started to fit the dose-dependence curves to sigmoidal functions of the type:


(2) $$\begin{array}{l} G_{\left(c_{p}\right)}=\frac{G_{\max}}{1+{\left(\frac{c_{p}}{MIC_{50}}\right)}^{m}} \end{array}$$

where c_p_ is the peptide concentration, G is the growth, m the slope, and G_max_ is the maximal growth of the bacteria. In a subsequent step the additive curves (CI = 1) are simulated, which can then be compared to the observed dose-dependent bacterial killing when both peptides are present in the mixture (103). Notably during the transition, the normalized bacterial density (y-axis) is particularly sensitive to even small changes in peptide concentration. Because of the steep dependence of bacterial growth on peptide concentration a small error in peptide concentration (along the x-axis) translates into a pronounced standard deviation when the growth is measured in replicates [i.e., a big error bar in y-axis; cf. (103)].

**Figure 2:**
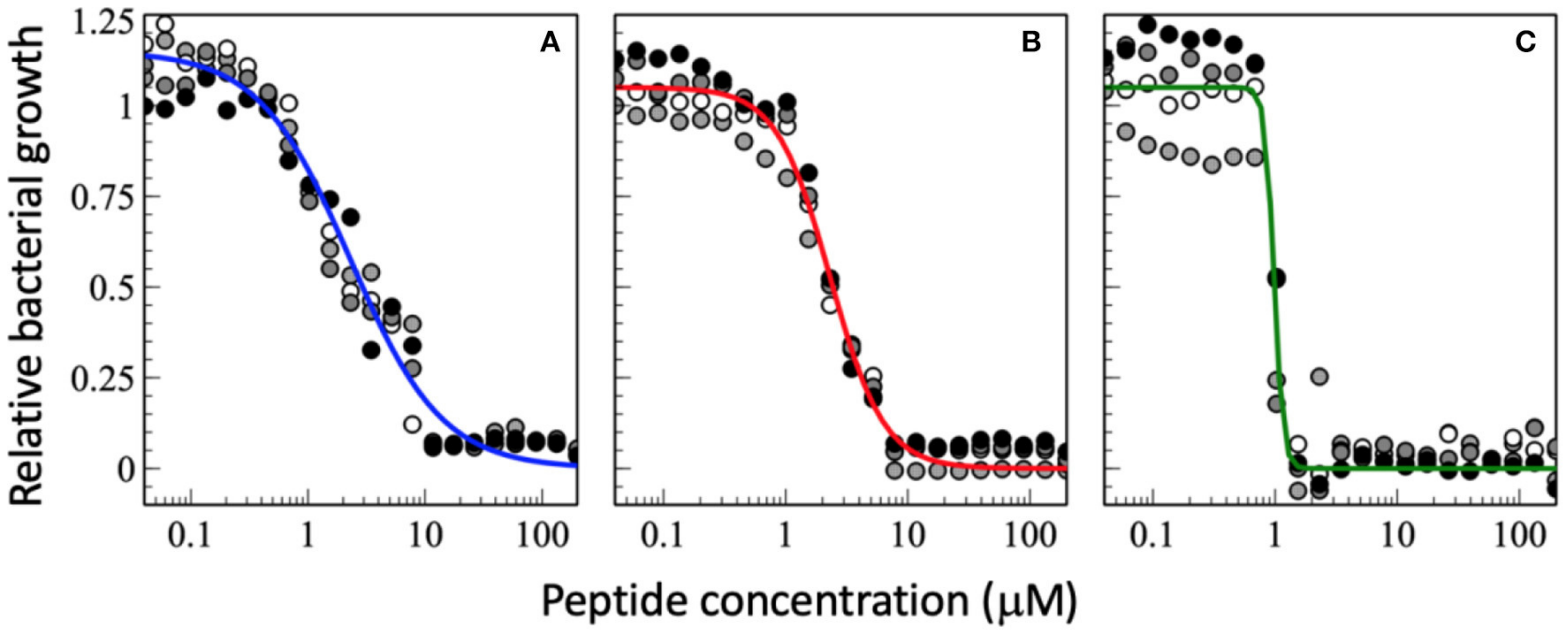
Dose-response curve of relative bacterial growth in the presence of **(A)** magainin 2a, **(B)** PGLa, and **(C)** the equimolar mixture of both. Bacterial supensions in MH medium (8.3 x 10^5^ CFU/mL) are added to a serial dilution of peptides and the optical density at 600 nm is recorderd after an 18 h inclubation at 37°C. The experiments were performed on 96-well microplates (F-bottom sterile non-treated polystyrene, Thermo Scientific Nunc A/S, Roskilde, Denmark). Starting from a 200 μM peptide concentration a sequential dilution series was performed with a dilution factor of 1.5 in 22 steps yielding final peptide concentrations ranging from 200 to 0.040 μM (after addition of bacteria). Each condition was done in quadruplet and each experiment is represented by different symbols in the plots (i.e., white, gray, and black circles represent a different experiment). The sequential dilution series were normalized to the bacterial growth without treatment on the same plate. The data shown have not been published before.

Whereas, in case of a steep dose-response the slope *m* of the sigmoidal cannot be calculated from a 2-fold dilution series and was initially chosen arbitrarily (103) it turns out that this cooperativity index bears additional information of interest once more data points around the MIC_50_ reveal interesting differences between the peptides. Figure 2 and Table 2 present so far unpublished data and a more elaborate scheme for the investigation of antimicrobial activities and synergism. Instead of the often applied 2-fold dilution series more data points are included (1.5 dilution series) thus revealing the slope m of the transition.

**Table 2:** This table shows the fit parameters of the blue, red, and green curves of Figure 2.

**Peptide**	**MIC_**50**_ (mM)**	**m**	**G_**max**_**
magainin 2a	2.31 ± 0.52	1.11 ± 0.10	1.15 ± 0.07
PGLa	2.32 ± 0.04	1.98 ± 0.09	1.05 ± 0.06
mix (1:1)	0.98 ± 0.05	11.8 ± 5.3	1.06 ± 0.12

*The data shown have not been published before*.

Four individual dilution series were fitted to Equation (2), from which averages for the MIC_50_, the slope *m*, and G_max_ were calculated and these were used to calculate the fits represented by the continuous lines in blue, red, and green.

It has been shown that at sub-MIC only a fraction of bacteria is killed and their debris including anionic polymers from the cell interior neutralizes much of the peptide added (153, 154). As a consequence, with some delay the survivors catch up in such laboratory suspensions and may reach the full cell density (151). Given the complete coverage of bacteria with peptides at the cell densities used in standardized antimicrobial activity assays, it is unlikely that the fractional killing is due to statistical variations in peptide density. Therefore, it has been suggested that fractional killing is a consequence of phenotypic variations of single cells which makes them more or less susceptible to the antibacterial activity of AMPs (153). The same authors have estimated that under sub-MIC conditions shortly after peptide addition a relatively small number of bacteria persists in a well thus such fluctuations potentially become apparent.

**Figure 3:**
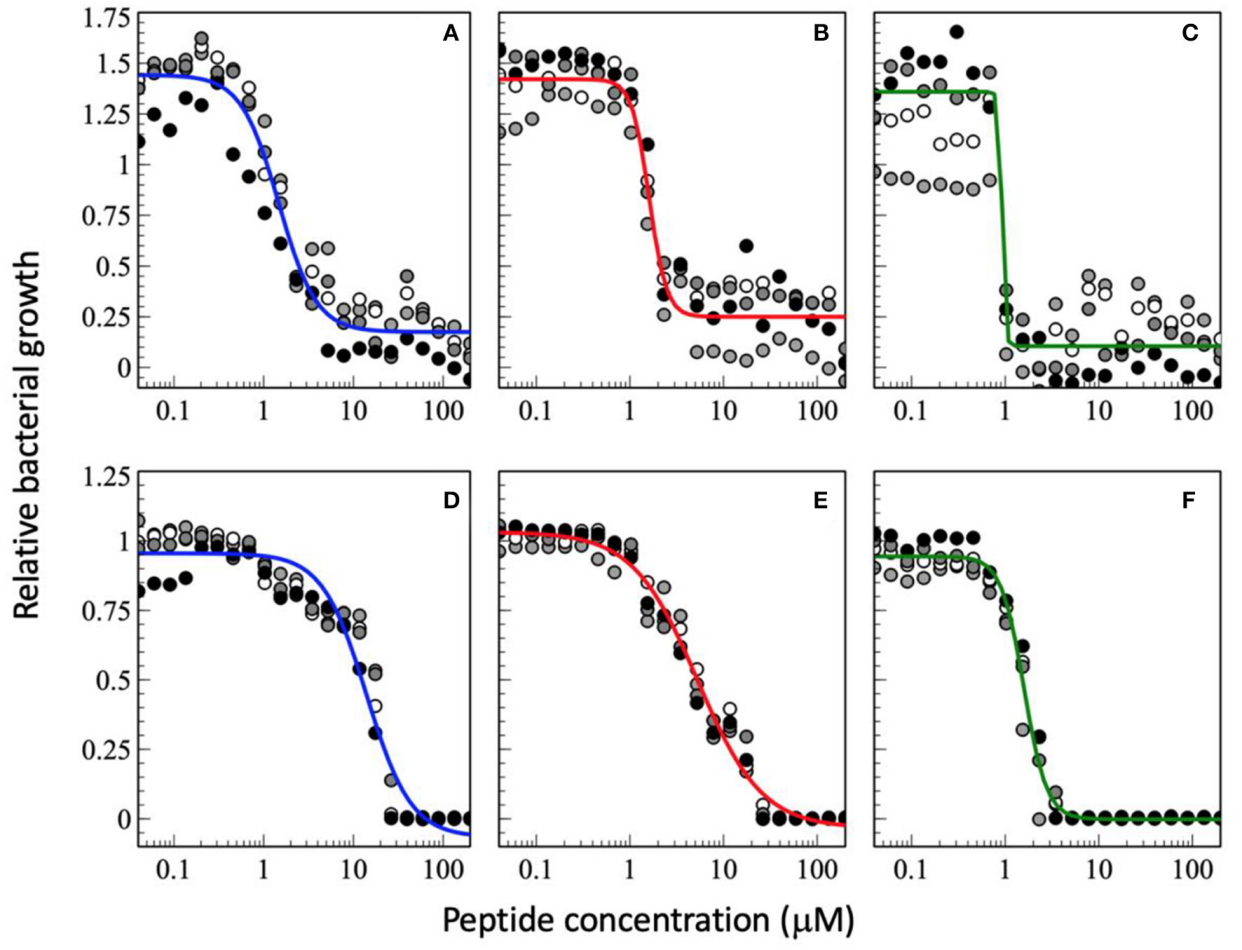
Dose-response curve of relative bacterial growth in the presence of antimicrobial peptides as a function of inocculum. The peptides were added to a bacterial supension in MH medium at low inocculum of 1.46 x 10^2^ CFU/mL **(A–C)** or at high inocculum at 1.51 x 10^7^ CFU/mL **(D–F)** and the optical density at 600 nm recorderd after an 18 h inclubation at 37°C. The peptides tested are magainin 2 **(A,D)**, PGLa **(B,E)**, and the equimolar mixture of both **(C,F)**. The experimental conditions are those of Figure 2. The data shown have not been published before.

Therefore, in the experiments shown in Figures 2, 3 the incubation of bacteria after addition of peptide was kept to 18 h which prevents that bacteria that are delayed in their growth by the peptides can catch up to full density. It is also interesting to note that the values G_max_ > 1 indicate that at low peptide concentrations and low to standard inoculum (<10^6^ CFU/mL) addition of peptide stimulates the bacterial growth.

Interestingly the slope *m* in the presence of PGLa is different from that of magainin (Figure 2). Most strikingly for the synergistic mixture the onset of activity is not that different but it has the deepest slope which brings the MIC_100_ from about 10 μM for the individual peptides to about 1 μM for the mixture. This 10-fold synergistic enhancement when the MIC_100_ is taken as an indicator decreases to a factor of 2.3 when the 50% killing is taken into consideration, i.e., the midpoint of the transition (Figure 2). Thus, a quantitative evaluation of synergy not only depends on the detailed experimental conditions but also the very details of how bacterial killing or growth are evaluated.

Because near the MIC_50_ only a fraction of the bacteria is killed the survivors are selected and take over, which may cause resistance development (153). It has been suggested that AMPs have a high Hill coefficient which explains why bacteria are less prone to develop resistance development (23). Here we show that the synergistic magainin 2/PGLa mixture exhibits even steeper slopes and hence adds an additional advantage (Figures 2, 3). It is possible that because the peptides affect bacterial subpopulations differently, there are less survivors that can fill the gap. The data also suggest that at least part of what is considered the synergistic interaction between the peptides is related to the steeper slope of the dose-response.

In a general sense *m* means some sort of cooperative or anti-cooperative behavior (i.e., non-linearity) (155) and in the case of a cooperative coefficient indicates that more than one molecule is required for a given activity. This can happen through oligomerization, but it could also mean that several peptides have to come into proximity on the bacterial surface (79), or that they form loosely packed lipid-peptide supramolecular structures (69). On the other hand, the slope of the curve can flatten should aggregation occur in solution at high peptide concentrations.

When the cell density is increased the MIC_50_ increases (Figures 3D–F, so far unpublished data). This is well-known phenomenon and has been associated with peptide adsorption to cell debris including anionic biopolymers and membranes (153, 156). Furthermore, cooperativity, especially of PGLa is reduced when the CFU per ml are increased. This probably reflects a larger phenotypic variability of cells with differences in AMP susceptibility (153). Furthermore, the synergy factor obtained by comparing the MIC_50_ values increases to 4.8 whereas it remains around 10 when the MIC_100_ are considered. Because at very high inoculum the peptides distribute on many more cells it may be more difficult to reach a high enough density to kill (154, 157, 158). Thereby, one may speculate that at high cell density the increased binding affinity and the mesophase structures when the two peptides are added conjointly (69) help to reestablish local hot spots and synergy. In this context it is noteworthy that the peptides have been shown to redistribute unevenly on the bacterial surface (159). Alternatively, it may also be possible that at high cell densities the ratio of surviving subpopulations increases thus it is easier to replenish the bacteria killed by a single antibiotic. This phenomenon would be less apparent when the combined action of two peptides keeps killing a large fraction of bacteria. Clearly, more experiments are needed to elaborate on these observations.

Interestingly, the best MICs are in the 1 μM range which agrees with observations made previously (103). As a consequence the synergistic factor is usually small for peptides that exhibit already high antimicrobial activity when investigated alone (103). This is also observed for the experimental series at a low CFU of 1.46 x 10^2^ CFU/mL where the synergy factor by comparing the MIC_50_ is 1.7 but again higher for the MIC_100_ (Figures 3A–C, so far unpublished data). However, it was recently pointed out that peptides also stick to surfaces of the test equipment thus the available concentration is probably significantly lower especially at low peptide concentrations (158).

## Magainin 2—PGLa Mixtures Show Synergistic Enhancement in Model Membranes

Such synergistic enhancements in antibacterial assays can occur through specific interactions (49, 160) but also when one compound helps the antimicrobial effector to reach its target (161). Thereby the PGLa/magainin 2 case is of particular interest because it works in pure lipid model membranes where the mode of action can be studied in biophysical detail (26, 119, 122, 162, 163). When the release of fluorophores from liposomal preparations was investigated these were made of well-defined lipid compositions thus revealing interesting lipid dependences.

First of all negative charges are important to assure a high local peptide concentration close to the membrane surface and thereby and increased membrane association (130, 134). Whereas, in the absence of negatively charged lipids the membrane partitioning coefficients of AMPs are in the 10^3^ M^−1^ range they apparently increase by more than an order of magnitude due to negative membrane surface charges (66). In some cases the association of antimicrobial peptides seems solely driven by electrostatic attraction until charge compensation is achieved (122, 135). This observation explains the lack of structuration when the peptides were investigated by optical techniques in dilute suspension of zwitterionic liposomes (122, 164). Electrostatic effects also contribute to their selectivity in killing of bacteria which exhibit a negative surface over healthy eukaryotic cells which appear electrically neutral to the outside (129–133). Therefore, when association and pore formation to zwitterionic membranes is very low the peptides may be too dilute in the membrane to interact with each other (119).

**Figure 4:**
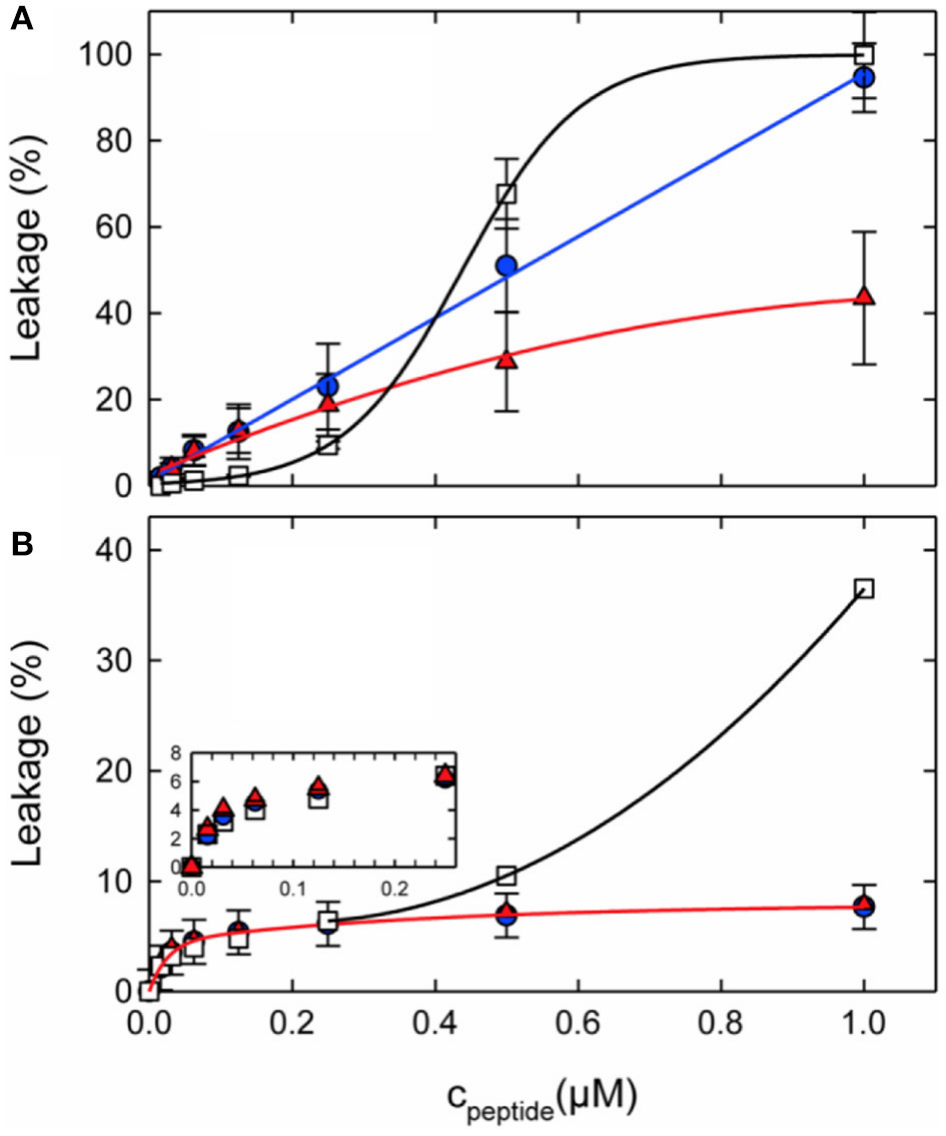
Calcein leakage from 100 nm unilamellar vesicles made from **(A)** POPC/POPG (3:1 mol/mol) or **(B)** POPE/POPG (3:1 mol/mol) as a function of peptide concentration. L18W-PGLa (blue circles), magainin 2a (red triangles), or their equimolar mixture (open squares) were added to 50 μM lipid. The lines were added to guide the eye. Taken from Leber et al. (119).

When fluorophore release and peptide synergy are investigated in a P/L ratio-dependent manner it becomes clear that a quantitative evaluation of synergy also depends on the detailed conditions of the experiment (Figure 4). For example, inspection of Figure 4B shows the absence of synergy at low peptide concentrations, but this value continuously increases at ≥0.4 μM. Notably either the amount of leakage after a few minutes (102, 119, 162) or the initial leakage rate (45, 163) have been taken as indicators of synergy.

From a combination of fluorescence-based biophysical experiments Matsuzaki and co-workers suggested early on that the rate of pore formation is slower for magainin, but the openings are more stable than those of PGLa (162). Thereby, synergism arises from fast pore formation and moderate stability. A more recent paper by Heerklotz and co-workers suggests that the combination of making large enough pores that are well distributed among the liposomes causes synergism in vesicle dye release experiments (165). According to these models the size of the vesicles or of the bacterial cells and the heterogeneity of the peptide distribution have an effect on the observed synergism (165).

## On Definitions to Quantitatively Define Synergistic Activity

A quantitative comparison of synergistic enhancement is further complicated not only due to differences in the experimental systems investigated but also by the different definitions used to quantitatively compare the activities of the mixture and individual peptide solutions. For example, Matsuzaki and co-workers compare the P/L ratio where 50% of the dye is released (162): $=\frac{\frac{P}{L_{50,\;calculated}}}{\frac{P}{L_{50,\;observed}}}$.

In this definition the calculated values are the average of the P/L_50_ ratios obtained from the individual peptides. This approach is similar to the calculated MICs published by Glattard et al. (103).

In contrast, rather than comparing the peptide concentration needed to reach a defined functional activity (e.g., 50% leakage) Zerweck et al. use the (variable) leakage values (*L*) at a fixed 100 μM lipid concentration and a fixed P/L. Typically conditions are chosen where a good range of activities is observed (i.e., 3 μM peptide for PE/PG/CL 72/23/5 and 0.6 μM for PC/PG 3/1). From this data a synergy factor is calculated according to SF $=\frac{L_{\left(P+M\right)}}{L_{\left(P\right)}+L_{\left(M\right)}}$.

Furthermore, from the same team the P/L of the mixture was twice that of the individual peptides in one publication (102) but kept constant in a prior paper (163). Finally, Leber et al. use “Peptide Synergy” Σ = 1/SF at the “highest peptide concentration” (cf. Figure 4 showing data up to 1μM) and 50 μM lipid (119). The total peptide concentration when peptides are tested individually is half of that of the mixture. This definition of synergy is based on the assumption that a heterodimer complex forms (G. Pabst, personal communication). While all of these definitions have their justification comparing the data quantitatively becomes impossible. For example, for PE-rich membranes the values include Σ =0.4 (corresponding to SF=2.5) (119) and SF = 22 (102). Furthermore, the PGLa-driven calcein release from PC/PE/PS 2:5:3 vesicles requires 25 to 43-fold less PGLa when 3.7 μM magainin are present (50 μg/ml lipid in 0.5 M NaCl, 10 mM PIPES, pH 7) (164). At this concentration magainin exhibits no activity when added alone (164).

## Correlating Synergism With Structural Investigations and Lipid Composition

Structural investigations were performed to develop a model how the peptides interact and thereby enhance their antimicrobial/pore-forming activities. When studied by solid-state NMR methods PGLa and magainin are both aligned along the surface of membranes when these carry at least one unsaturation per phospholipid (98, 99, 116, 166), including *E. coli* lipid extracts (103). Thus, in such membranes the helix topology resembles those of magainin or PGLa alone, i.e., in the absence of the other peptide (89, 98). In contrast, when magainin 2/PGLa mixtures are studied in fully saturated lipid bilayers magainin remains oriented along the membrane surface whereas PGLa adopts transmembrane alignments (98, 99). However, it should be noted that unsaturations are abundant in biological membranes thus a mechanism for synergistic antibacterial activities should consider an alignment of both peptides along the bilayer surface (98, 101, 103).

Not only the lipid fatty acyl chain but also the lipid head group composition seems of considerable importance for the synergistic enhancement to develop. Notably, in recent investigations it was demonstrated that for lipids with intrinsic negative curvature such as PE or PC/cholesterol the pore forming activity of the individual peptides is reduced when compared to PC/PG membranes. However, much of the activity was restored when adding the peptide mixture. This behavior results in a significant synergetic enhancement of activities in PE/PG but not in PC/PG membranes (Figure 4). In this context it should be noted that the antimicrobial activities of PGLa or magainin individually are much lower in PE/PG than in PC/PG, thereby synergetic enhancement in the bacterial membrane mimetic abolishes the differences observed for the individual peptides when investigated in membranes of different composition (cf. Figure 4 at 1 μM). This agrees with observations made when the antimicrobial activity of magainin and PGLa as well as derivatives thereof were investigated and the highest synergy was observed for peptides with intrinsically low activities (103).

Notably, a fluorescence quenching investigation has revealed the formation of mesophase structures along the membrane surface and correlated diffusion of both peptides (69). Fluorescence quenching occurs when the fluorophores, which were added to the amino-terminus of either magainin 2 or PGLa, approach each other within the nm range, i.e., closer than expected from a statistical distribution (104). These experimental observations are in-line with a recently published MD simulation of the peptide mixture where in stacked membranes a string of interacting peptides and lipids has been observed (117). Interestingly, the observed mesophases and diffusion correlation come along with a much increased membrane partitioning of the peptides when the mixture is investigated in PE/PG but not in the presence of PC/PG membranes (69). Because the membrane disruptive properties of magainin 2 extends over several layers of lipids (ca 0.8 nm in diameter) (112, 113) the formation of mesophases with interpeptide distances <1 nm (104) suggests a concerted action of several peptides in destabilizing the membrane (69). In this context is it interesting that microscopic imaging approaches have revealed an uneven distribution of antimicrobial peptides with preferential association with curved regions of the bacterial membrane such as pole caps or the septum of dividing cells (47, 77).

During early work on magainin/PGLa synergism little attention was given to the lipid compositions of the model systems investigated. With new data pointing to important effects of lipids exhibiting intrinsic negative membrane curvature it is of interest to review earlier publications. Indeed, the very early investigations were performed in PC/dicetylphosphate membranes of different (PC/DCA ratios) where dicetylphosphate is a small negatively charged “head group” made of phosphate carrying two C16 chains (26, 45). Thus, in a lipid membrane the molecule exhibits an inverted cone shape which is associated with negative curvature [cf. (122)]. Furthermore, experiments were performed with azolectin a soy bean lipid mixture which contains PC, PE, and PIs (45). Synergistic enhancements of fluorophore release were also observed with BBPS or egg-PC membranes (162), and thereby do not seem to fit the requirements of negative curvature observed by Leber et al. (119). However, it should be noted that the synergy factors published in this work are only 3.5 and thereby relatively low when compared to the antibacterial tests presented in the same paper where a factor of 8 was observed (162). For EYPC very high P/L ratios of 0.57, 0.14, and 0.05, respectively, were required to measure the S value. Thereby, although typically 100 nm vesicles are used for most of the dye release experiments (102, 119, 162) the detailed conditions are otherwise quite different and the values may not easily be compared, in particular as the dose response curves are not linear (Figure 4).

## Biophysical Measurements OF PGLa—Magainin Interactions in Membranes

Spectral changes that occur upon membrane partitioning can be used to derive membrane association constants of polypeptides (69, 135, 167, 168). From such data the interaction between the membrane associated peptides can be derived including quantitative estimates of the energies involved. However, these values are only valid in the context of an assumed molecular model. In this manner favorable PGLa-magainin interaction energies were obtained in egg-PG membranes (162). Notably the quantitative evaluation of the data depends how many peptides are assumed to be involved in the interaction process. Energies for homo- and hetero-dimer formation have also been published for fully saturated membranes where PGLa exhibits a transition into the TM state (163). Therefore, this analysis probably includes many energy contributions (101, 169) which remain unimportant when synergy occurs between peptides that reside along the membrane surface of a bacterial membrane (103).

In a more recent investigation association of the two peptides with LUVs made of POPE/POPG 3/1 at pH 7 as a bacterial membrane mimetic were investigated by Isothermal Titration Calorimetry (ITC) (70) indicating strong membrane association with apparent membrane association constants in the 10^6^ M^−1^ range (apparent stoichiometry P/L ≈ 1.7 mole%). Whereas, the membrane association of magainin and PGLa is characterized by an endothermic reaction enthalpy an additional exothermic contribution becomes apparent when the peptide mixture is titrated. Thus, these ITC data reveal an additional model-independent ΔH in the range of −2 kcal/mole in the magainin 2/PGLa mixture when compared to the peptides individually. This reaction enthalpy could be due to e.g., vesicle agglutination as observed by DLS experiments conducted on the same system (70). Peptide-driven intermembrane interactions were also apparent by a reduction in the bilayer repeat distance of mechanically oriented membranes (110, 117). Furthermore, the formation of loosely packed supramolecular assemblies also contributes to an energetic contribution in particular as these have been shown to correlate with an order of magnitude increased membrane affinity in the presence of POPE/POPG (69). Notably, when the proximity and thereby the interactions between magainin 2 and PGLa when associated to POPE/POPG 3/1 or POPC/POPS 3/1 membranes was tested by fluorescence spectroscopy a FRET effect was observed at high P/L ratios where close encounters of the peptides happen statistically (170). However, this effect diminishes and disappears when diluting the peptide with more lipid indicating that possible interactions between the peptides are rather weak.

## Sequence Specificity of Magainin 2—PGLa Synergy

In order to define key structural elements of the synergistic interactions and residues that may be involved in the magainin 2—PGLa interactions the peptide sequences have been modified and tested. When the F16W or E19Q modifications of magainin 2 were studied for fluorophore release from egg PC/PG (1:1) liposomes a reduced synergistic activity was observed whereas the F5W alteration did not exhibit any effect (162). Introducing a positive charge at position 19 much abolished the synergistic interactions (102). In contrast, neutralizing the magainin 2 carboxyterminus by amidation has no effect on its synergism with PGLa (102, 103, 170, 171) although the antimicrobial activity of the peptide is increased (171, 172). Similar observations were made with the hydrophobic face of the magainin 2 helix (173).

Furthermore, when PGLa is modified the synergistic activity with magainin 2 is maintained even when a proline is added to the N-terminus and a negative charge to the C-terminus of PGL (171). When key residues of PGLa were searched exchanging the positive charges of lysines 15 and 19 by glutamines abolish synergistic enhancement whereas a more moderate reduction is measured for K5E and K12E (102) and L18W is tolerated (119, 162).

Such potential electrostatic interactions between the membrane-associated peptides have been suggested early on and are visualized in coarse grain and all-atom MD simulations even though the membranes used in the older simulations do not fulfill the criteria of a physiological composition (116, 117, 174, 175).

Furthermore, residues G7, G11, and L18 of PGLa have been shown important (102). Glycines 7 and 11 form a GxxxG motif which has been suggested to promote dimerization in highly apolar environments (176). However, the PGLa localization at the membrane interface (98, 103) may not be suitable for stable PGLa homodimer interactions. A detailed structural analysis of the peptide mixture in a lipid bilayer is required to resolve such ambiguities. In summary, the charges located at the carboxyterminal ends of the peptides have the strongest effects on synergy and an important role was also associated with G7, G11, and L18 of PGLa which needs further investigation.

## Lessons Learned From Peptide Dimers

In previous publications the question how the oligomerization of peptides along the membrane surface influences activity was already discussed (122, 177). In the context of models proposed for the mechanisms of synergistic enhancement the comparison with covalently linked dimers is also of interest. Therefore, in a first step possible interactions between membrane-associated PGLa and magainin 2 were tested by preparing peptides carrying GGC extensions and cross linking experiments (178). In PC/PG (1/1) bilayers parallel dimers preferentially form (178), therefore, dimers linked through C-terminal GGC extensions were prepared and investigated. When compared to the same amount of unmodified peptides in a mixture all of, the (PGLa-GGC)_2_ and (magainin-GGC)_2_ homodimers as well as the magainin-GGC/PGLa-GGC heterodimer were all more active in calcein release experiments from POPE/POPG 3/1 liposomes (119). However, only the PGLa-homodimer and the PGLa-magainin heterodimer, but not the individual peptides or their mixture, showed significant dye release from POPC/Cholesterol 3/1 liposomes (119). When the antimicrobial activities of the wildtype magainin/PGLa mixture and the dimer of the GCC-derivatives are compared to each other a complication arises from the fact that the GGC extensions itself make the monomeric peptides more active (103, 149). Furthermore, a dimer linked through a carboxyterminal lysine extension was considerably more active than the monomer whereas the amino-terminal linkage through glutamic acid has no effect (179).

Furthermore, a cystine-linked magainin 2 dimer has been shown more active in membrane permeabilization and antimicrobial activities (180). From the ensemble of data it seems that the increased activity of the dimers is based on an increased membrane perturbation by the larger peptide aggregates rather than due to a particular structure of the PGLa-magainin 2 dimer (122, 180, 181). In line with such a model is the observation that the homotarsinin homo-dimer (2 x 24 residues) is more active in antibacterial assays than the corresponding amount of monomer (181).

Another dimer that has been studied is distinctin a heterodimer which forms a compact dimer of two dimers in solution which has been suggested to protect the protein from proteolytic digestion (182) thereby resulting in a slightly increased antimicrobial activity of the dimer when compared to the monomers (183). Its solution structure unfolds in the presence of membranes thus the 25-residue chain 2-helix partitions into the membrane parallel to the membrane surface. In contrast, the 22-residue chain 1 associates only loosely with the membrane (92, 184). Thereby the dimer acts similar to a monomeric linear cationic peptide.

## Model for Synergistic PGLa—Magainin Interactions

In order to develop a model for antimicrobial synergism focus should be on structural data obtained in membranes carrying unsaturations and PE head groups such as they occur in bacterial membranes. Structural data indicate that the highly cationic peptides adopt amphipathic α-helical conformations and that these peptides are aligned parallel to the membrane surface (98, 101, 103). MD simulation and diffraction data show that the peptides partition into the interface of POPE/POPG 3/1 membranes with the large hydrophobic face of PGLa being inserted somewhat deeper than magainin 2 (103, 116, 117). Electrostatic interactions involving the dipolar charge distribution along the peptides (102, 103, 162) and interactions involving anionic lipids (104) and/or anions of the surrounding buffers (185) help in the formation of nematic phase arrangements along the membrane surface (Figure 1).

**Figure 5:**
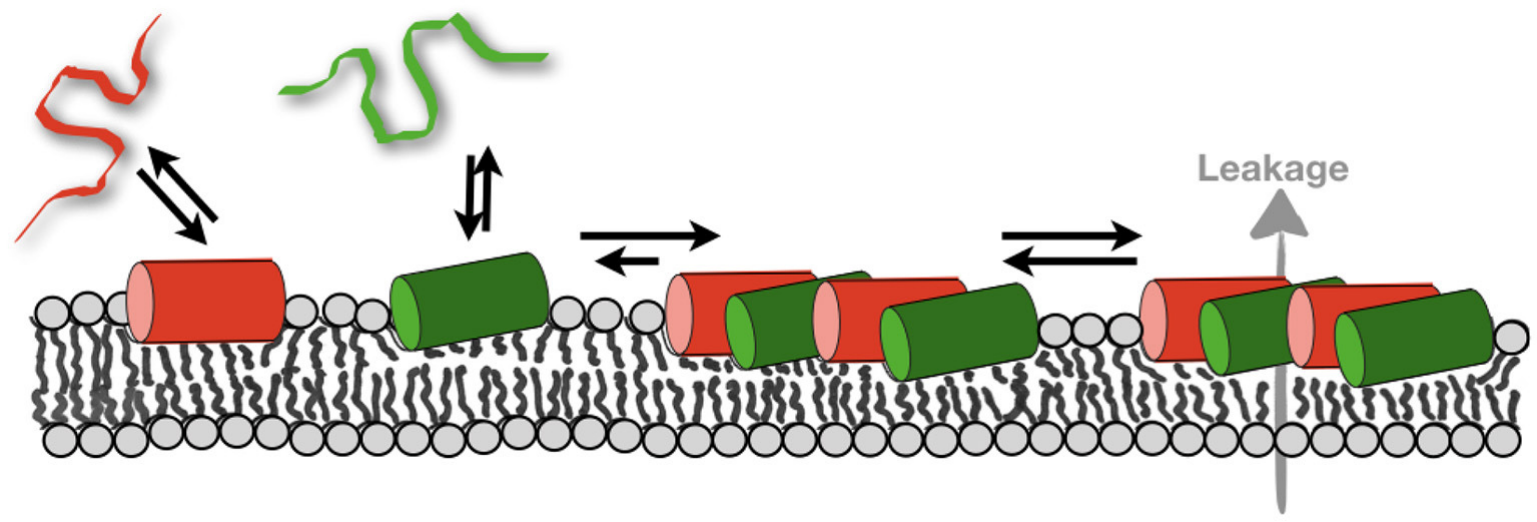
Schematically illustrates the membrane partitioning equilibria of magainin 2 (red) and PGLa (green). The formation of mesophases deletes the pool of monomeric peptides thereby more peptide can bind. As a consequence, the total amount of membrane-associated peptide increases and supramolecular structures that facilitate leakage form (69).

Furthermore, when the peptides partition into the membrane interface they have been shown to have a large disordering effect on the fatty acyl chains of the surrounding lipids (101). Interestingly, such and related changes in the membrane packing and structure have been postulated to result in lipid-mediated interactions over several molecular layers (186–188). The peptides disturb the finely tuned equilibrium of van-der Waals, hydrogen bonding and electrostatic interactions, entropic contributions of the lipid fatty acyl chains and of the membrane-associated water, that assure the lipid bilayer packing into well-defined supramolecular arrangements. Therefore, it is possible that the pronounced lipid disordering observed in the presence of magainin 2, PGLa, and the mixture constitutes an important driving force to bring peptides into closer proximity (Figure 5). Thereby new supramolecular arrangements of the lipids and peptides form which have been detected by fluorescence quenching techniques and MD simulations (69, 117). Notably, zones of high peptide density have also been observed when bacteria were imaged (159) but these measurements work on very different length scales and it is not clear if these observations correlate. Notably, the formation of supramolecular arrangements within POPE/POPG membranes and the correlation observed between PGLa and magainin 2 comes along with an order of magnitude increase in membrane partitioning of the peptides (Figure 5). The much-increased membrane affinity due to the presence of the other peptide by itself could explain the synergistic enhancement of activities, but the formation of peptide-lipid mesophases results in high local peptide concentrations which can also involve a modulation of activities.

While extensive studies have been performed to define the range of pathogens and tumor cells susceptible to be killed by magainin antimicrobial peptides or the lack of toxicity against healthy human (or frog) cells (61, 62, 172, 189) much less data on toxicity or antimicrobial action are published about the synergistic mixture of PGLa and magainin 2 (26, 44, 190). For the individual peptides selectivity of bacteria over healthy eukaryotic cells has been explained by the negatively charged surface of bacteria and a high negative inside membrane potential of their plasma membrane assuring a high density of polycationic peptides at the bacterial membrane (129, 191). Furthermore, eukaryotic cells are protected from membrane lysis by these peptides due to the presence of cholesterol (62, 192). Because in this model the preferential killing of bacteria over healthy human cells is driven by the physico-chemical properties of the membrane similar considerations should also be applicable to the peptide mixtures. In this context it is notable that the synergistic enhancement of activities works for PE-rich (bacterial) membranes but not when this lipid is replaced by PC (eukaryotic membranes). Thereby, such biophysical findings suggest that the therapeutic window of these peptides potentially increase when added in combination.

## Author Contributions

EG and DJ performed experiments. BB wrote the manuscript. All authors contributed to writing the manuscript and in the preparation of Figures.

## Conflict of Interest

The authors declare that the research was conducted in the absence of any commercial or financial relationships that could be construed as a potential conflict of interest.

## Abbreviations

AMP: antimicrobial peptide
ATR FTIR: attenuated total reflection Fourier transform infrared
CFU: colony-forming unit
CD: circular dichroism
CL: cardiolipin
DCP: dicetylphosphate
DOPC, 1: 2-dioleoyl-*sn*-glycero-3-phosphocholine
DOPG, 1: 2-dioleoyl-*sn*-glycero-3- phospho-(1′-*rac*-glycerol)
GUV: giant unilamellar vesicle
ITC: isothermal titration calorimetry
LUV: large unilamellar vesicle
MD: molecular dynamics
MIC: minimal inhibitory concentration
NMR: nuclear magnetic resonance
PC: phosphatidylcholine
PE: phosphatidylethanolamine
PG: phosphatidylglycerol
PS: phosphatidylserine
POPC: 1-palmitoyl-2-oleoyl-*sn*-glycero-3-phosphocholine
POPE: 1-palmitoyl-2-oleoyl-*sn*-glycero-3-phosphoethanolamine
POPG: 1-palmitoyl-2-oleoyl -*sn*-glycero-3- phospho-(1′-*rac*-glycerol)
POPS: 1-palmitoyl-2-oleoyl-*sn*-glycero-3-phosphoserine
SMART, Soft Membranes Adapt and Respond: also Transiently
TM: transmembrane.
